# Improved plant biomass production under low nitrogen conditions through conditional accumulation of the second messenger, guanosine tetraphosphate, in chloroplasts and mitochondria

**DOI:** 10.3389/fpls.2024.1524665

**Published:** 2025-01-13

**Authors:** Mina Goto, Takanari Nemoto, Kazuma Sakoda, Atsushi Sakurai, Sousuke Imamura, Shinji Masuda

**Affiliations:** ^1^ Department of Life Science and Technology, Institute of Science Tokyo, Yokohama, Japan; ^2^ Space Environment and Energy Laboratories, NTT Corporation, Musashino-shi, Japan

**Keywords:** arabidopsis, biomass, chloroplast, mitochondria, nitrogen starvation, ppGpp

## Abstract

To enhance plant biomass production under low nitrogen conditions, we employed a method to artificially and temporarily accumulate the bacterial second messenger, guanosine tetraphosphate (ppGpp), to modify plastidial or mitochondrial metabolism. Specifically, we fused a chloroplast or mitochondrial transit-peptide to the N-terminus of the bacterial ppGpp synthase YjbM, which was conditionally expressed by an estrogen-inducible promoter in *Arabidopsis*. The resulting recombinant *Arabidopsis* plants exhibited estrogen-dependent ppGpp accumulation in chloroplasts or mitochondria and showed reduced fresh weight compared to wild type (WT) plants when grown on agar-solidified plates containing a certain amount of estrogen. This finding aligns with the previous study indicating that plastidial ppGpp levels can influence plant biomass production. When the recombinant plants were grown in the soil with estrogen and low nitrogen-containing water at specific time intervals, they exhibited greater fresh weight than WT plants. These results suggest that the conditional accumulation of ppGpp in not only chloroplasts, but also in mitochondria can lead to improved plant biomass production in soil with low nitrogen applications.

## 1 Introduction

In recent years, global population growth has led to a demand for increased plant biomass as a source of food and energy (Becker and Fanzo, 2023; Bodirsky et al., 2015). Most soils on the earth have low nitrogen concentrations in nature, and are not suitable for plant growth (Lal et al., 2022). A nitrogen fertilizer application is one of the methods to achieve favorable biomass production of plants in agriculture (Bodirsky et al., 2015), but the continuous and large-amount application results in eutrophication of rivers and oceans due to the nitrogen inflow from the soil (Xia and Yan, 2023). On this background, it will be necessary to establish genetic conditions for improving plant growth with creating plant individuals that can grow well even with low nitrogen applications.

In plants, the stringent response is known to have a key function in their growth under low nitrogen conditions. The stringent response is one of the most important nutrient starvation response in bacteria, which was originally discovered in *Escherichia coli* a half century ago (Cashel, 1969; Potrykus and Cashel, 2008). In the stringent response, unusual nucleotide, guanosine 5’-diphosphate 3’-diphosphate (ppGpp) functions as a second messenger that control transcription, translation, DNA replication and other enzyme activities in response to various environmental changes. In *E. coli*, ppGpp is synthesized by the two distinct enzymes RelA and SpoT, and hydrolyzed by SpoT (Potrykus and Cashel, 2008). Recent studies have shown that ppGpp-dependent stringent response is conserved not only in bacteria, but also in eukaryotes including plants (Field, 2018; Masuda, 2012; Tozawa and Nomura, 2011). In plant cells, all ppGpp synthases, called RelA-SpoT homologs (RSHs), are localized in plastids (Givens et al., 2004; Masuda et al., 2008; Sugliani et al., 2016; Tozawa et al., 2007), suggesting that the stringent response in plants controls plastid gene expression and metabolisms. Interestingly, overaccumulation of ppGpp in plastids impact on plant growth and biomass. Specifically, a recombinant *Arabidopsis* that constitutively accumulates ppGpp by ~3-fold increases fresh weight compared to WT (Maekawa et al., 2015). On the other hand, *Arabidopsis* mutant constitutively accumulating ppGpp by ~10-fold decreases fresh weight of shoots compared to WT (Sugliani et al., 2016). This suggests that the amount of ppGpp accumulated in chloroplasts could be a determinant of individual biomass. In addition, the increase in biomass due to RSH overexpression has been reported to be more pronounced under low nitrogen concentrations (Goto et al., 2022, 2024; Honoki et al., 2018). These results suggest that the control of ppGpp accumulation possibly increases plant biomass production even under low nitrogen conditions through modifying the stringent response intensity.

The mitochondrion is another organelle in plant cells that is thought to have originated through the endosymbiosis of an ancestral α-proteobacterium and retains bacterial metabolic processes similar to those in chloroplasts (Gross and Bhattacharya, 2011). However, all RSHs are localized exclusively in chloroplasts and not in mitochondria as mentioned above, suggesting that ppGpp does not function in mitochondria in plant cells. Although ppGpp has the potential to function in mitochondria, the impact of ppGpp accumulation in mitochondria has not yet been tested.

In this study, we constructed transgenic *Arabidopsis* by which ppGpp accumulation in chloroplasts could be arbitrarily changed. We also targeted the ppGpp synthase to mitochondria to induce the stringent response in the organelle. The recombinant plants were then used to clarify the relationship between ppGpp levels and plant biomass. The obtained results indicated that ppGpp accumulation in both chloroplasts and mitochondria could enhance plant growth and biomass in soil under low nitrogen conditions.

## 2 Materials and methods

### 2.1 Plants and growth conditions

The Columbia ecotype of *Arabidopsis thaliana* was used as a wild-type (WT) strain. Plants were grown on 0.8% agar-solidified half concentration Murashige and Skoog (MS) medium at 23°C in growth chambers with continuous light illumination (40–50 μmol photons m^–2^ s^–1^). After 14 days, plants were transferred to the soil (vermiculite:promix ratio of 1:1) and grown under continuous light illumination of 40–50 μmol photons m^–2^ s^–1^. Water containing 0.03 mM KNO_3_ and different amount of estrogen (0, 0.1 or 1.0 μM) was applied to the soil. β-estradiol (estrogen) (Sigma-Aldrich) was dissolved in dimethyl sulfoxide (DMSO) at a concentration of 1.0 and 10 mM, and diluted by 10,000-times with water for 0.1 or 1.0 μM estrogen conditions, respectively. As for 0 μM estrogen conditions, water containing 0.01% DMSO was used.

### 2.2 Construction of transgenic plants

Transgenic *Arabidopsis* lines expressing chloroplast- and/or mitochondrion-targeted YjbM were constructed as follows. First, DNA fragments encoding the chloroplast transit signal of *recA* (At1g79050) and the mitochondrion transit signal of γ-ATPase (At2g33040) were separately amplified by PCR using the following primer pairs: 5’-GAAGCTAGTCGACTCTAGCCATGGATTCACAGCTAGTCTTG-3’ and 5’-TCCCACTGTTTGTCATCCATGTCGCGATCGAATTCAGAACTG-3’ for the chloroplast signal, and 5’-GAAGCTAGTCGACTCTAGCCATGGCAATGGCTGTTTTCCGT-3’ and 5’-TCCCACTGTTTGTCATCCATGTCGCGGTTCTTAACACTCTTCATGCG-3’ for the mitochondrion signal. Genomic DNA from WT *Arabidopsis* was used as a template. Previously, we constructed an estrogen-inducible YjbM-expressing plasmid construct (Ihara and Masuda, 2016) using the pER8 plasmid (Zuo et al., 2000). An inverse PCR was performed with the pER8-*yjbM* plasmid (Ihara and Masuda, 2016) as a template to amplify the *yjbM*-coding region along with the plasmid backbone of pER8 using the primer pair 5’-ATGGATGACAAACAGTGGGAA-3’ and 5’-GGCTAGAGTCGACTAGCTTCA-3’. The DNA fragments encoding the chloroplast and mitochondrial transit peptides were then separately mixed with the inverse PCR fragment and connected using In-Fusion cloning reagent (Clontech). The resulting pER8-based plasmids, encoding *yjbM* fused with the chloroplast and mitochondrial target signals, were named pER8-cpYjbM and pER8-mtYjbM, respectively. After verifying the correct sequences of the inserted DNA, pER8-cpYjbM and pER8-mtYjbM were separately introduced into the WT *Arabidopsis* using standard *Agrobacterium*-dependent transformation methods. Homozygous lines were isolated in the second or third generations and used for further analysis.

### 2.3 Western blotting and determination of ppGpp

Protein samples from each plant were electrophoresed through an SDS-PAGE gel (12% w/v acrylamide) and electroblotted onto a polyvinylidene fluoride membrane (GE Healthcare). Protein concentrations were determined by Lowry assay kit (BioRad), and the same amount of the protein was loaded in each lane. Membrane-bound proteins were immuno-probed with antibodies against anti-FLAG M2 (Thermo Fisher 31430), and detected using ECL Plus Western Blotting Detection kit reagents (GE Healthcare). ppGpp quantification was done by a modified method described previously (Ihara et al., 2015; Ito et al., 2020).

### 2.4 Statistical analysis

All experiments were designed and performed with at least three independent replicates. The collected data were calculated and the graphs and charts constructed using Microsoft Excel and *R* software. The statistical differences between the transgenic lines and WT were confirmed using the Dunnett test or *t*-test. Other details such as sample sizes are shown in each figure legend.

## 3 Results

### 3.1 Construction of transgenic *Arabidopsis* conditionally accumulating ppGpp

**Figure 1:**
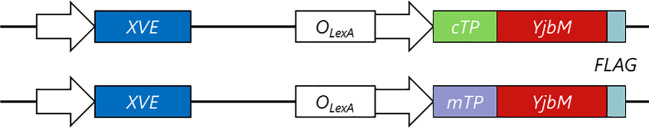
A schematic model showing estrogen-inducible cTP-YjbM and mTP-YjbM expressing transfer-DNA constructs. A chimeric transcription activator, XVE, was created by fusing the DNA-binding domain of the bacterial repressor LexA, the transactivating domain VP16, and the regulatory region of the human estrogen receptor (Zuo et al., 2000). The transactivating activity of XVE, whose expression was controlled by a constitutive promoter, can be strongly regulated by estrogen application. DNA fragments encoding FLAG-tagged cTP-YjbM and mTP-YjbM were separately placed downstream of the LexA-recognizing operator (*O_LexA_*).

The ppGpp synthase activity of most ppGpp synthases, such as *E. coli* RelA and SpoT, is regulated at posttranslational levels (Potrykus and Cashel, 2008). However, the *Bacillus subtilis* ppGpp synthase YjbM, which consists solely of a ppGpp synthase domain without any activity control domains, is known to continuously synthesize ppGpp upon expression (Nanamiya et al., 2008). In this study, YjbM was modified with an additional chloroplast or mitochondrial transit peptide (cTP or mTP, respectively) and the FLAG tag at its N-terminus and the C-terminus, respectively, and the recombinant genes were separately placed downstream of an estrogen-inducible promoter (**Figure 1**). We used the cTP from *Arabidopsis* RecA and the mTP from γ-ATPase, which were previously employed for specific targeting of a fluorescent protein to chloroplasts (Köhler et al., 1997) and mitochondria (Masuda et al., 2003), respectively. These constructs were separately introduced into WT *Arabidopsis* via *Agrobacterium*-mediated transformation. Given that all RSHs in *Arabidopsis* target and function within chloroplasts, but not mitochondria (Maekawa et al., 2015; Masuda et al., 2008; Mizusawa et al., 2008), ppGpp-dependent metabolic control may not naturally occur in mitochondria of plant cells. However, both chloroplasts and mitochondria are believed to have originated from the endosymbiosis of an ancient cyanobacterium and α-proteobacterium, respectively (Gross and Bhattacharya, 2011; Stadnichuk and Kusnetsov, 2021). Therefore, if YjbM is artificially targeted to mitochondria, it could potentially synthesize ppGpp and subsequently influence mitochondrial metabolism. As a result, several recombinant WT plants expressing cTP-YjbM or mTP-YjbM were successfully obtained.

**Figure 2:**
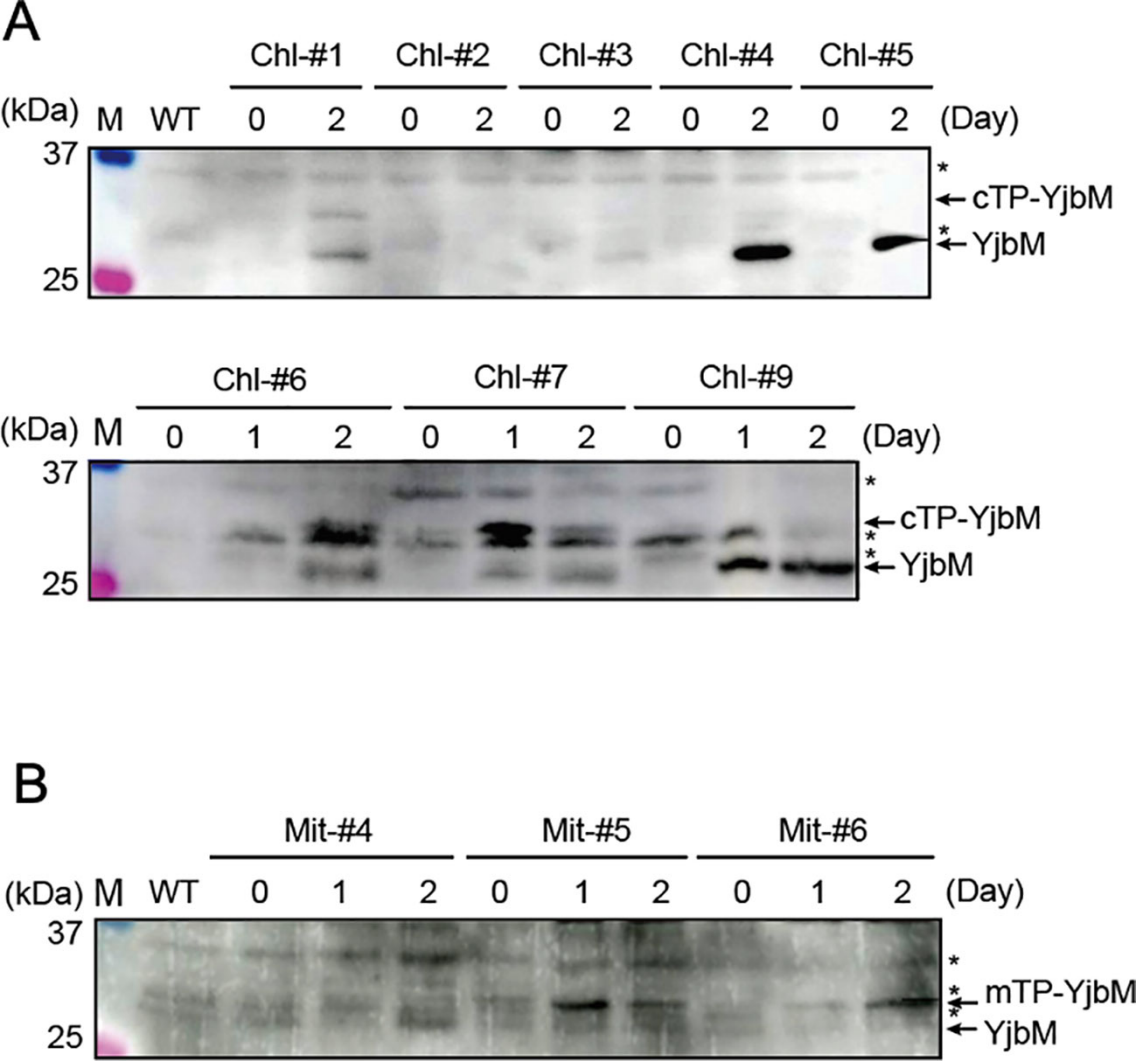
Western blot analysis to check the expression of YjbM localized to chloroplasts **(A)** and mitochondria **(B)**. Plants were grown on agar-solidified plates without estrogen for 14 days, then transferred to new plates containing 1.0 μM estrogen for 1 or 2 days. Protein extracts from leaf tissues were separated by SDS-PAGE and subjected to Western blotting using an anti-FLAG antibody. Asterisks indicate non-specific bands. M, size markers.

To confirm whether the recombinant YjbM is expressed and correctly targeted to chloroplasts or mitochondria in each mutant, western blotting was performed using an anti-FLAG antibody against total protein in each plant line after estrogen treatment. For the analysis, plants were grown on agar-solidified plates in the absence of estrogen for 14 days, and then transferred to new plates containing 1.0 μM estrogen. Shoots were harvested one or two days after estrogen treatment and subjected to western blotting. The molecular weights of FLAG-tagged cTP-YjbM, mTP-YjbM, and transit peptide-cleaved YjbM, as deduced from their amino acid sequences, are 33, 32, and 27 kDa, respectively. Seven cTP-YjbM expressing lines, designated Chl-#1, Chl-#3, Chl-#4, Chl-#5, Chl-#6, Chl-#7, and Chl-#9, exhibited a 27 kDa band after one or two days of estrogen treatment (**Figure 2A**), indicating that cTP-YjbM is successfully targeted to chloroplasts in these lines, although the levels of the targeted protein varied among them. Chl-#1, Chl-#6, and Chl-#7 also showed a 33 kDa band after estrogen treatment (**Figure 2A**), suggesting that a portion of cTP-YjbM remains unprocessed and accumulates in the cytosol in these lines. In contrast, two mTP-YjbM expressing lines, designated Mi-#4 and Mi-#5, displayed a 27 kDa band after one or two days of estrogen treatment (**Figure 2B**), indicating that mTP-YjbM is targeted to mitochondria in these lines. The targeted YjbM levels were higher in Mi-#4 than in Mi-#5. Additionally, Mi-#5 and Mi-#6 exhibited a 32 kDa band after estrogen treatment (**Figure 2B**), suggesting that some mTP-YjbM remains unprocessed and accumulates in the cytosol in these lines.

### 3.2 Accumulation of ppGpp in plants expressing YjbM localized to chloroplasts and mitochondria

**Figure 3:**
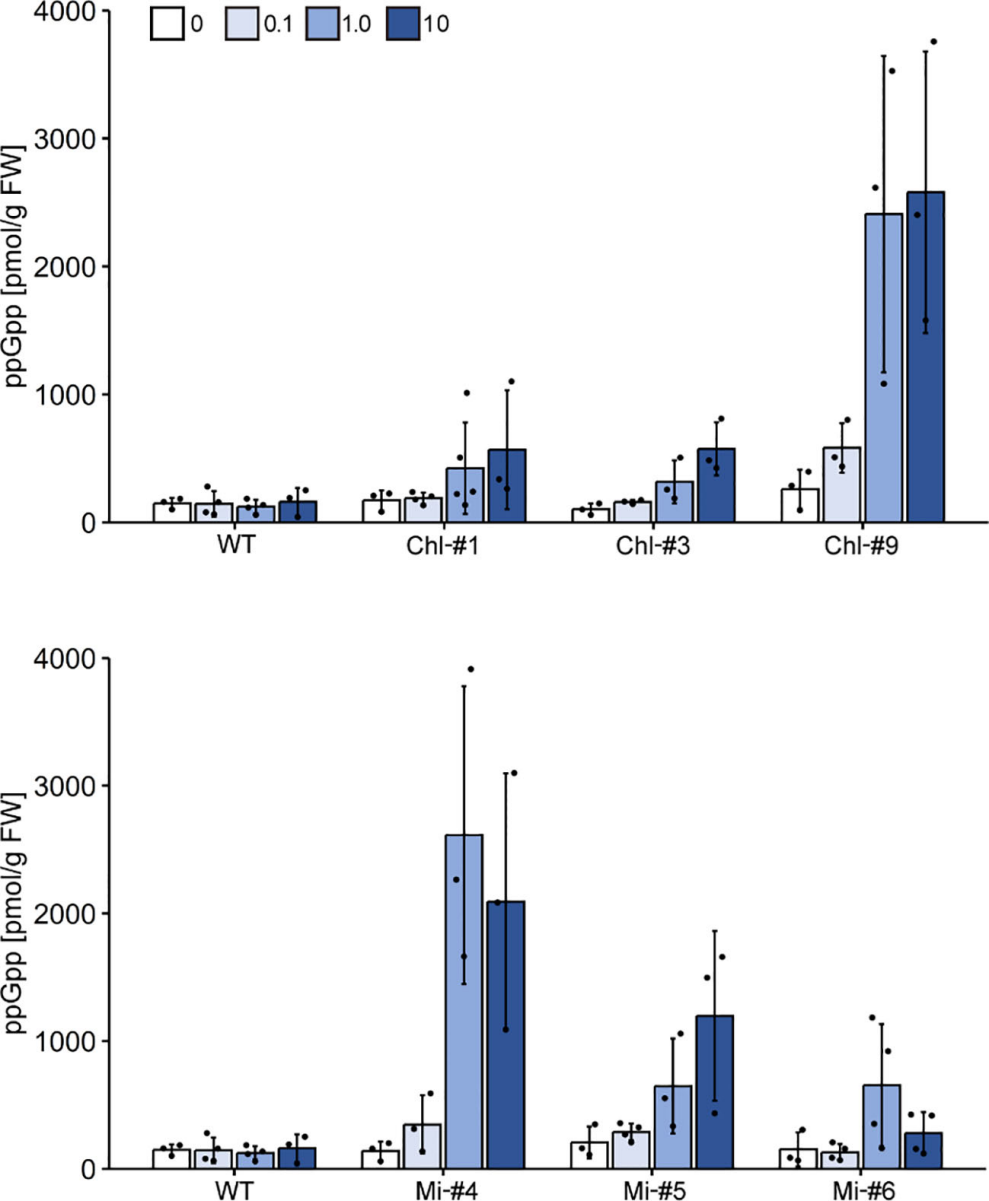
Accumulation of ppGpp upon estrogen treatment. Plants were grown on agar-solidified plates without estrogen for 14 days, then transferred to new plates containing different concentrations of estrogen (0, 0.1, 1.0, or 10 μM) for 2 days. ppGpp levels in shoot tissues were then measured. Data are presented as means ± S.D. (n = 3–4).

To determine the amount of ppGpp accumulated in each line, ppGpp quantification was performed. For the analysis, plants were grown on agar-solidified plates in the absence of estrogen for 14 days, then transferred to new plates containing different concentrations of estrogen (0, 0.1, 1.0, or 10 μM). We quantified ppGpp levels in three cTP-YjbM-expressing lines, Chl-#1, Chl-#3, and Chl-#9, as well as in three mTP-YjbM-expressing lines, Mi-#4, Mi-#5, and Mi-#6, which exhibited different levels of targeted YjbM (**Figure 2**). The results showed that ppGpp accumulation increased in an estrogen concentration-dependent manner, and the amount of ppGpp varied across each line (**Figure 3**). Specifically, Chl-#1 and Chl-#3 exhibited small ppGpp accumulation in the presence of 1.0 μM and 10 μM estrogen. In contrast, Chl-#9 showed higher ppGpp levels than WT in the presence of >0.1 μM estrogen. Given that the expression level of cTP-cleaved YjbM in Chl-#9 was higher than in Chl-#1 and Chl-#3 (**Figure 2A**), ppGpp levels appear to be correlated with the amount of YjbM targeted to chloroplasts.

Among the mTP-YjbM-expressing lines, Mi-#4 accumulated the highest levels of ppGpp, with over 20 times more ppGpp than WT at estrogen concentrations >1.0 μM. Mi-#5 and Mi-#6 displayed higher ppGpp levels than WT at 1.0 μM estrogen, although Mi-#6 showed no difference in ppGpp levels compared to WT at 10 μM estrogen. Similar to the cTP-YjbM expression lines, ppGpp levels were well correlated with the amount of YjbM targeted to mitochondria; the expression level of transit peptide-cleaved YjbM increased in the order of Mi-#4, Mi-#5, and Mi-#6 (**Figure 2B**). It should be noted that the levels of non-cleaved mTP-YjbM remaining in the cytosol did not correlate with the amount of ppGpp. Specifically, the levels of non-cleaved YjbM decreased in the order of Mi-#4, Mi-#5, and Mi-#6 (**Figure 2B**), suggesting that YjbM with the transit peptide was not functional in the cytosol.

### 3.3 Phenotype of plants expressing YjbM localized to chloroplasts and mitochondria

**Figure 4:**
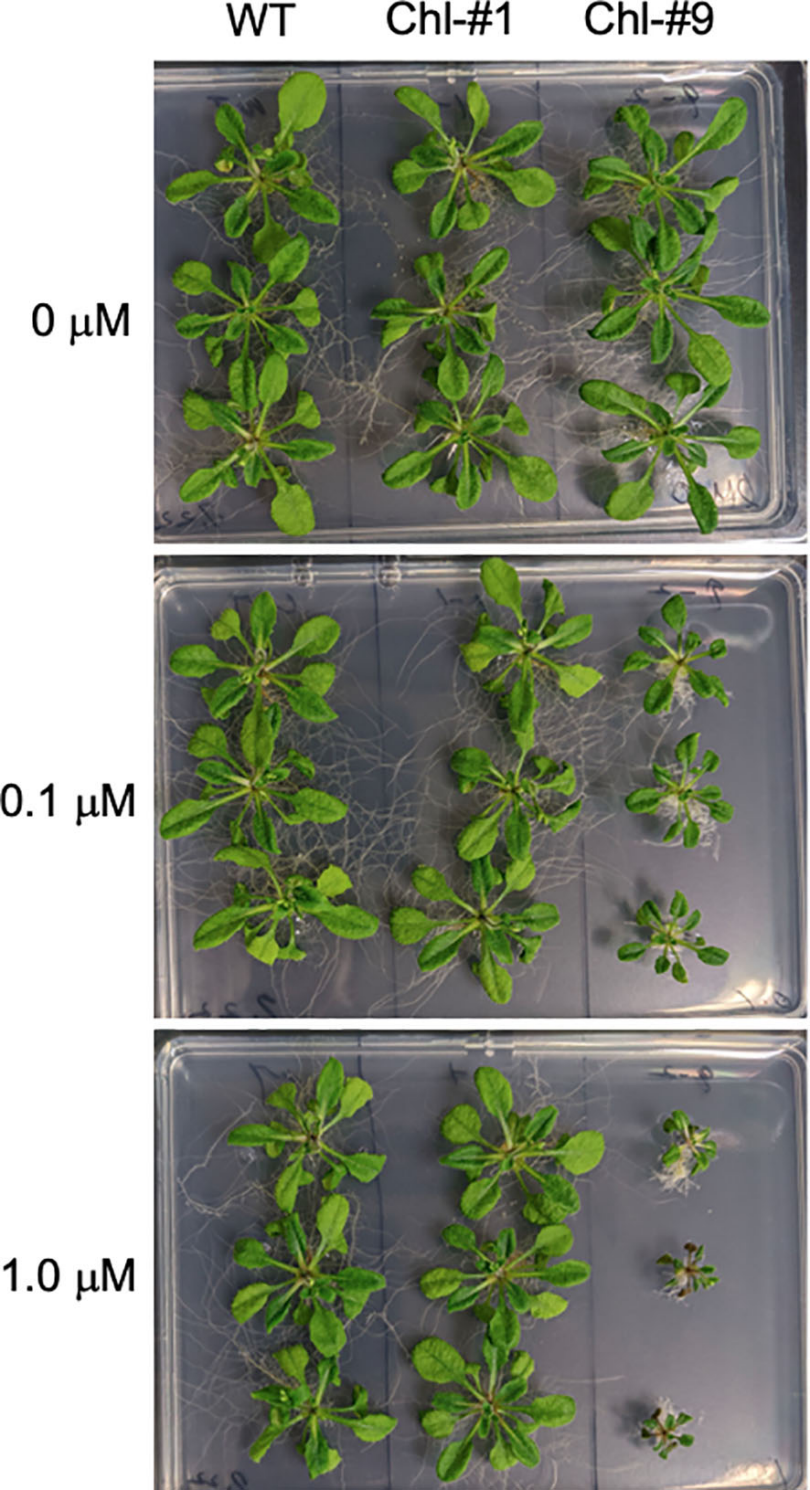
Appearance of Chl-#1 and Chl-#9 lines in plate experiments. Plants were grown on agar-solidified plates without estrogen for 14 days, then transferred to new plates containing different concentrations of estrogen (0, 0.1, and 1.0 μM) for 10 days.

**Figure 5:**
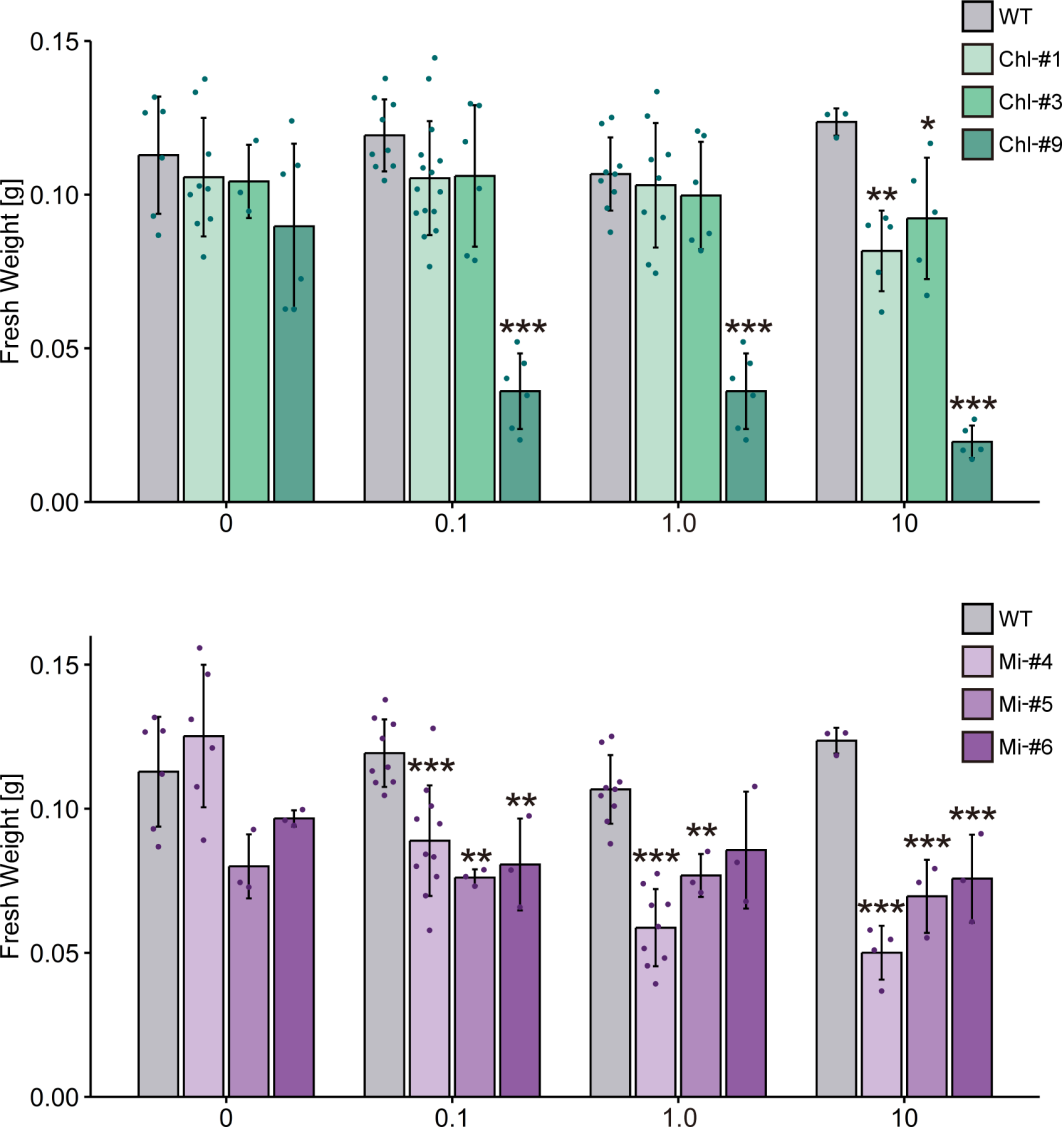
Fresh weight of transgenic plants expressing YjbM localized to chloroplasts (Chl-#1, Chl-#3, and Chl-#9) and mitochondria (Mi-#4, Mi-#5, and Mi-#6) in plate experiments. Plants were grown on agar-solidified plates without estrogen for 14 days, then transferred to new plates containing different concentrations of estrogen (0, 0.1, 1.0, and 10 μM) for 10 days. Data are presented as means ± S.D. (n = 3–11). Asterisks indicate significant differences compared to WT (p < 0.05; Dunnett test).

To examine the effect of ppGpp accumulation on plant growth, we grew transgenic lines of *Arabidopsis* on normal plates for 14 days, then transferred them to plates containing different concentrations of estrogen for 10 days. Some mutants exhibited smaller individual sizes compared to WT in the presence of estrogen (**Figure 4**), and fresh weight of all transgenic lines tested was significantly reduced in the presence of estrogen compared to WT (**Figure 5**). Specifically, among the transgenic lines expressing chloroplast-targeted YjbM, Chl-#9, which accumulated high amounts of ppGpp (**Figure 3**), showed a significant reduction in fresh weight to about half that of WT at an estrogen concentration of 0.1 μM. At concentrations above 1 μM, fresh weight of Chl-#9 was reduced to one-fifth that of WT (**Figure 5**). Phenotypically, new leaves showed white discoloration in the veins, and anthocyanin accumulation was observed on the underside of the leaves of Chl-#9 under 1.0 μM estrogen conditions (**Figure 4**). The Chl-#9 line could not germinate on plates containing >1.0 µM estrogen (**Supplementary Figure 1**). Additional phenotypes of the line included short roots. Mi-#4, which also accumulated high levels of ppGpp (**Figure 3**), had less than half the fresh weight of WT at an estrogen concentration of 1.0 μM (**Figure 5**).

**Figure 6:**
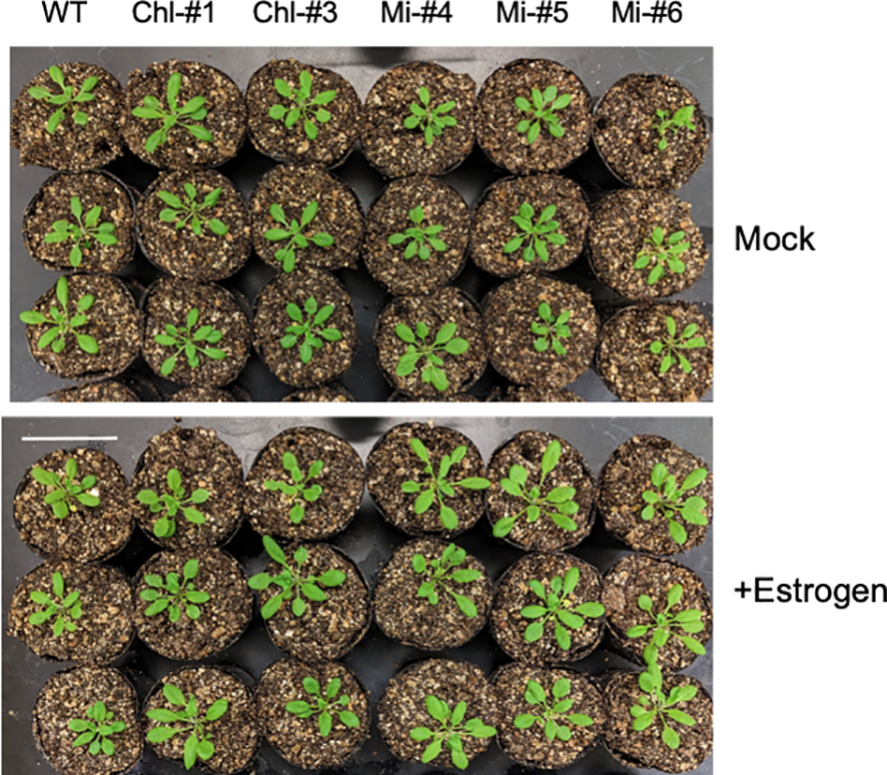
Appearance of transgenic plants expressing YjbM localized to chloroplasts (Chl-#1 and Chl-#3) and mitochondria (Mi-#4, Mi-#5, and Mi-#6) in soil experiments. Plants were grown on agar-solidified plates without estrogen for 14 days, then transferred to soil and further grown with water containing either 0.01% DMSO (Mock) or 1.0 μM estrogen with 0.01% DMSO for 10 days. Scale bar = 5 cm.

**Figure 7:**
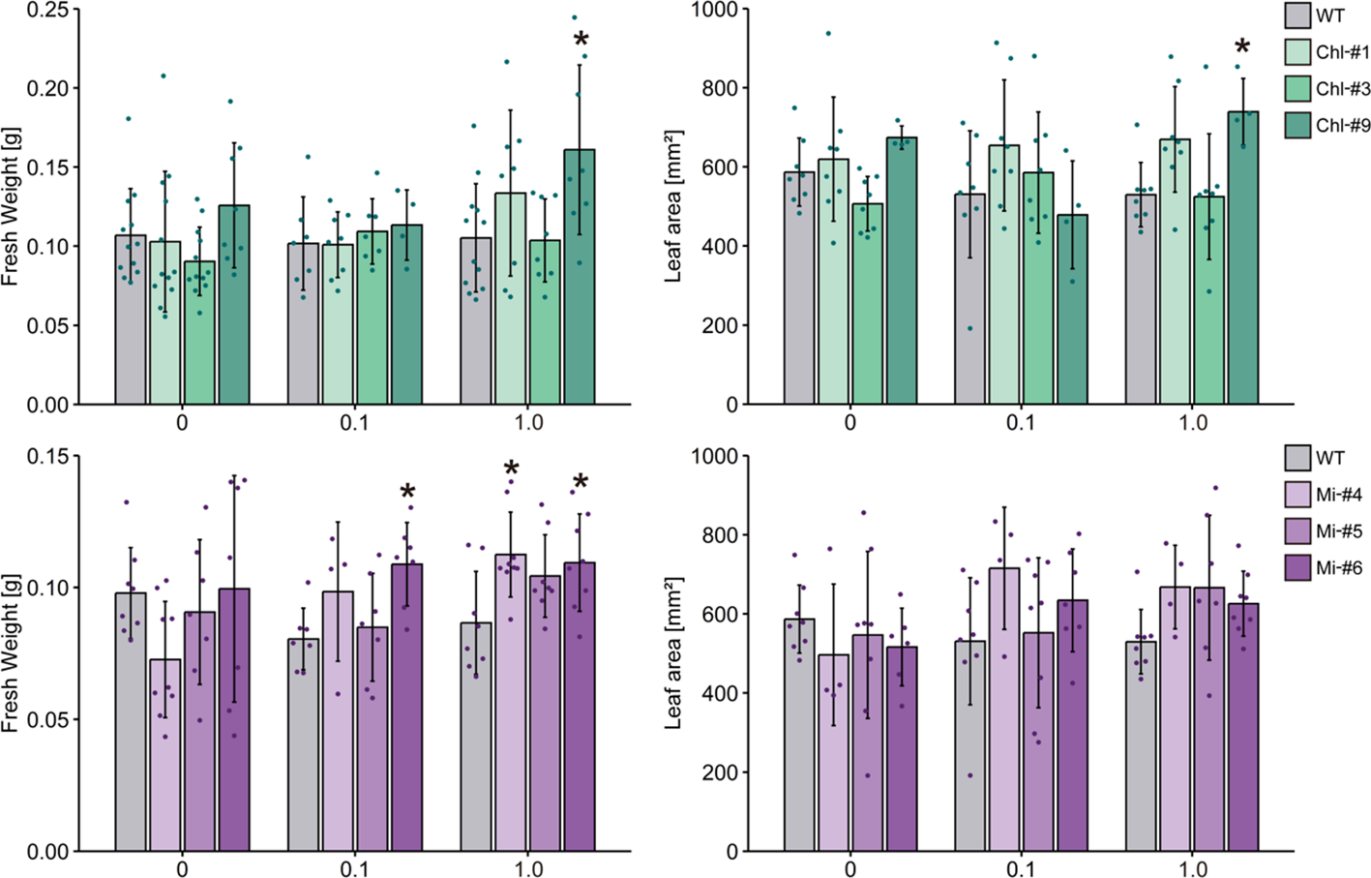
Fresh weight and leaf area of transgenic plants expressing YjbM localized to chloroplasts (Chl-#1, Chl-#3, and Chl-#9) and mitochondria (Mi-#4, Mi-#5, and Mi-#6) in soil experiments. Plants were grown on agar-solidified plates without estrogen for 14 days, then transferred to soil and further grown with water containing different concentrations of estrogen (0, 0.1, and 1.0 μM) for 10 days. The water also contained 0.01% DMSO. Data are presented as means ± S.D. (n = 3–8). Asterisks indicate significant differences compared to WT (p < 0.05; Dunnett test).

To obtain more information contributing to applied research aimed at increasing plant biomass production, we next examined the effect of estrogen-induced ppGpp accumulation on plant growth in soil. Plants were first grown on plates without estrogen for 14 days, then transferred to soil and watered with solutions containing 0, 0.1, and 1.0 μM estrogen. We used low nitrogen-containing soil (0.03 mM KNO_3_) to observe the impact of ppGpp accumulation on plant biomass (Goto et al., 2022, 2024). No significant effect of estrogen treatment on visible phenotype was observed in WT, Chl-#1 and Chl-#3 (**Figure 6**). On the other hand, shoot size of the transgenic lines expressing mitochondrion-targeted YjbM (Mi-#4, Mi-#5 and Mi-#6) seem to be larger than WT when plants were watered with estrogen (**Figure 6**). Statistical analysis indicated that Mi-#4 and Mi-#6 exhibited significantly greater fresh weight than WT under 1.0 μM estrogen conditions, with Mi-#6 also showing significantly greater fresh weight than WT under 0.1 μM estrogen conditions (**Figure 7**). Additionally, Chl-#9 showed significantly higher fresh weight and leaf area compared to WT under 1.0 μM estrogen conditions. Notably, these phenotypes were not observed under mock (DMSO) conditions (**Figure 7**).

Finally, we quantified ppGpp levels in plants grown in soil. For the analysis, plants were first grown on plates without estrogen for 14 days, then transferred to soil and grown for 10 days without estrogen. Plants were then watered with solutions containing 1.0 μM estrogen, grown for 2 days, and then harvested for ppGpp quantification. We first compared ppGpp levels of WT and the transgenic lines expressing chloroplast-targeted YjbM, which showed that there is no significant difference between WT and the transgenic lines in the ppGpp levels. Specifically, the measured values (pmol g^-1^ fresh weight) are as follows (sample size 4): 64 ± 20 (WT), 125 ± 92 (Chl-#1), 148 ± 38 (Chl-#3) and 94 ± 14 (Chl-#9) (*P* > 0.05, *t-*test). We also found that there is no significant difference in ppGpp levels between WT and the transgenic lines expressing mitochondrion-targeted YjbM; the measured values (pmol g^-1^ fresh weight) are as follows (sample size 4): 56 ± 26 (WT), 89 ± 28 (Mi-#4), 75 ± 31 (Mi-#5) and 96 ± 31 (Mi-#6) (*P* > 0.05, *t-*test). It should be noted that ppGpp is accumulated in the transgenic lines after 2 days from estrogen treatment on plates (**Figure 3**), suggesting that the effect of estrogen on ppGpp accumulation in the transgenic lines does not last long in soil.

## 4 Discussion

We successfully constructed *Arabidopsis* transgenic lines that can conditionally express chloroplast- or mitochondrion-targeted ppGpp synthase YjbM upon estrogen treatment. Using these transgenic lines, we demonstrated that ppGpp may function in mitochondria, as evidenced by the dwarf phenotype observed when ppGpp accumulated in this organelle. Specifically, Mi-#4, Mi-#5, and Mi-#6, which exhibited varying levels of ppGpp hyperaccumulation, showed reduced fresh weight compared to WT following estrogen treatment (**Figure 5**). Given that mitochondria are believed to have originated from the endosymbiosis of an ancient aerobic α-proteobacterium (Gross and Bhattacharya, 2011), gene expression and metabolic processes in mitochondria retain bacterial characteristics, which appear to be regulated by ppGpp. In bacteria, ppGpp binds competitively to certain GTP-binding proteins, including translation initiation and elongation factors (Kanjee et al., 2012), suggesting that homologous proteins in mitochondria could be ppGpp targets. Furthermore, ppGpp regulates activity of many enzymes involved in nucleotide metabolisms (Kanjee et al., 2012), indicating that those mitochondrial enzymes (Witte and Herde, 2019) might also be regulated by heterologously synthesized ppGpp. However, it should be noted that the accumulation levels of ppGpp and plant growth retardation did not show a clear negative correlation in the Mi-#4, Mi-#5, and Mi-#6 lines. All three lines exhibited similar plant growth retardation (**Figure 5**), despite variable levels of ppGpp accumulation in each line (**Figure 3**). This suggests that the effects of ppGpp on mitochondrial metabolism may become saturated at low ppGpp levels. Additionally, it cannot be completely ruled out that the transit peptide-fused YjbM (**Figure 2B**) catalyzes ppGpp synthesis in the cytosol, potentially influencing plant growth (Ihara and Masuda, 2016), even though the accumulation levels of ppGpp (**Figure 3**, bottom) and unprocessed mTP-YjbM (**Figure 2B**) did not show a correlation.

Reduction in fresh weight was also observed in transgenic lines expressing chloroplast-targeted YjbM (**Figure 5**), indicating that ppGpp accumulation in chloroplast is also negatively correlated with fresh weight. Specifically, Chl-#9, which exhibited ppGpp hyperaccumulation (~20-fold compared to WT) under 1.0 μM estrogen treatment (**Figure 3**), displayed a dwarf phenotype under these conditions (**Figure 4**). In contrast, Chl-#1, which did not show significant ppGpp hyperaccumulation under 1.0 μM estrogen treatment (**Figure 3**), did not exhibit the dwarf phenotype (**Figure 4**). These results suggest that ppGpp accumulation in chloroplasts negatively impacts plant growth. This hypothesis is further supported by the fact that an *Arabidopsis* RSH3-overexpression mutant, which showed a constitutive ~10-fold increase in ppGpp accumulation compared to WT, also exhibited lower fresh weight than WT (Sugliani et al., 2016). Conversely, we previously showed that another *Arabidopsis* RSH3-overexpression mutant, RSH3ox2, with a ~3-fold increase in ppGpp accumulation compared to WT, displayed greater fresh weight than WT (Maekawa et al., 2015). These findings suggest that relatively lower levels of ppGpp synthesis induced by estrogen may contribute to increased plant biomass, although this condition was not found in our plate experiments. Alternatively, the observed phenotype in RSH3ox2 might be due to the indirect effects of disrupting *rsh2* and *rsh3*, and/or the additional introduction of RSH3. However, some transgenic lines exhibited increased fresh weight compared to WT when grown in soil (**Figure 7**). This may be due to differences in estrogen stability between the two conditions. On plates, ppGpp is likely synthesized continuously because a certain level of estrogen is maintained under the sterile environment. In contrast, in soil, the presence of various microorganisms is thought to degrade estrogen, preventing the maintenance of a constant estrogen concentration. The gradual decrease in estrogen concentration likely reduced ppGpp accumulation, which may have positively impacted individual fresh weight. It has been reported that *Arabidopsis* WT accumulates ppGpp under nitrogen deficiency stress (Romand et al., 2022) as well as upon light-to-dark transition (Inazu et al., 2024; Ono et al., 2021), but this accumulation is only transient. Perhaps such a transient ppGpp accumulation in chloroplasts and even in mitochondria could contribute to plant fitness and increased biomass, while constant ppGpp accumulation is likely toxic.

The mechanism by which ppGpp, synthesized in chloroplasts or mitochondria, contributes to increasing plant biomass, particularly under nitrogen starvation, remains elusive. In *Arabidopsis*, ppGpp-deficient and ppGpp-accumulating mutants exhibited significant alterations in transcript and metabolite levels in whole tissues (Inazu et al., 2024; Ono et al., 2021; Romand et al., 2022), regulated through communication between the nucleus and the organelles. This suggests that ppGpp plays a role in the comprehensive regulation of metabolism in plant cells. One possibility is that ppGpp functions as a retrograde-signaling molecule, transported from organelles to the cytosol and/or nucleus to regulate enzyme activities at transcriptional, translational, and post-translational levels, either directly or indirectly. Further characterization of transgenic lines generated in this study will help elucidate the impact of artificial ppGpp accumulation on plant physiology.

## Data Availability

The raw data supporting the conclusions of this article will be made available by the authors, without undue reservation.
