# Supplementary material for: Improved plant biomass production under low nitrogen conditions through conditional accumulation of the second messenger, guanosine tetraphosphate, in chloroplasts and mitochondria
Source: Front Plant Sci. 2025 Jan 13;15:1524665. doi: 10.3389/fpls.2024.1524665 (PMC11770007; doi:10.3389/fpls.2024.1524665)
Supplement: Supplementary file 1 [file DataSheet1.pdf]

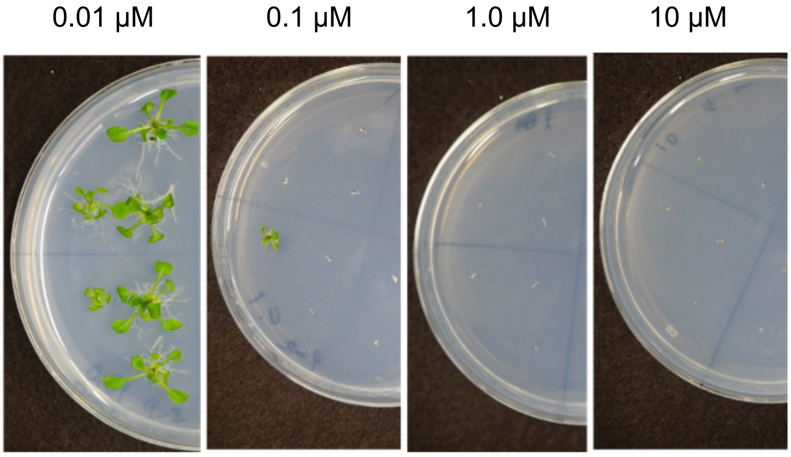


**Supplementary Figure 1** Growth of the Chl-#9 line on agar-solidified plants with different concentrations of estrogen (0.01, 0.1, 1.0, or 10 µM). Seeds were spread out on the plates and grown for 21 days.
